# Supplementary material for: Engineering a SARS-CoV-2 Vaccine Targeting the Receptor-Binding Domain Cryptic-Face via Immunofocusing
Source: ACS Cent Sci. 2024 Sep 17;10(10):1871–84. doi: 10.1021/acscentsci.4c00722 (PMC11503491; doi:10.1021/acscentsci.4c00722)
Supplement: Supplementary file 1 — oc4c00722_si_001.pdf [file oc4c00722_si_001.pdf]

Supporting Information

Engineering a SARS-CoV-2 Vaccine Targeting the Receptor-Binding Domain Cryptic-Face via Immunofocusing

Theodora U.J. Bruun, Jonathan Do, Payton A.-B. Weidenbacher, Ashley Utz, Peter S. Kim

Supporting information contains supplementary materials and methods, Fig. S1-S9, Table S1, and protein sequences.

SUPPLEMENTAL MATERIALS AND METHODS

Circular dichroism (CD). Samples for CD were prepared by buffer exchanging proteins into PBS (10 mM phosphate, 2.7 mM KCl, pH 7.4, Bioland Scientific LLC, Cat# PBS01-03) and then filtered through a 0.22-µm filter. The sample concentration was determined using a Nanodrop 2000 (ThermoFisher). CD spectra was collected using a Jasco J-815 CD Spectrometer sampling every 0.5 nM between 260 nM and 180 nM. Three accumulations were collected and averaged from each sample and the signal from a buffer only sample run under the same conditions was substracted from each sample run. The data was converted to units of mean residue ellipticity and plotted in GraphPad Prism 9.5.1. Data are reported until the voltage of the buffer sample reached 400 V.

Neutralization assay with SARS-CoV-2 Omicron variants. SARS-CoV-2 Omicron pseudotyped lentiviruses were produced as described in the main text based on the Omicron sequence (sequence ID: UFO69279.1) with specific amino-acid substitutions listed below for Omicron BA.1, BA.2, BA.4/5, BQ.1, BQ.1.1, and XBB.1.5. Neutralization assays were performed in the same way as described in the main text with the exception that instead of doing serial dilutions with heat-inactivated mouse antisera, neutralization of lentiviruses was measured at a final antisera dilution of 1/160 for all mice. The neutralization assay was run in duplicate for all mice against all viruses and infection percent was normalized to the signal in cells only wells (0% infection) and virus only wells (100% infection). The average neutralization for the 5 mice in each immunization group RWT, R11K, R11K-PMD, R11K-CL was calculated and plotted in GraphPad Prism 9.5.1.

SARS-CoV-2 Omicron Strain	Amino acid substitutions or deletions
**BA.1**	A67V, Δ69-70, T95I, Δ142-Δ144, Y145D, Δ211, L212I, G339D, S371L, S373P, S375F, K417N, N440K, G446S, S477N, T478K, E484A, Q493R, G496S, Q498R, N501Y, Y505H, T547K, D614G, H655Y, N679K, P681H, N764K, D796Y, N856K, Q954H, N969K, L981F
**BA.2**	T19I, Δ24-26, A27S, G142D, V213G, G339D, S371F, S373P, S375F, T376A, D405N, R408S, K417N, N440K, S477N, T478K, E484A, Q493R, Q498R, N501Y, Y505H, D614G, H655Y, N679K, P681H, N764K, D796Y, Q954H, N969K
**BA.4/5**	T19I, Δ24-26, A27S, G142D, V213G, G339D, S371F, S373P, S375F, T376A, D405N, R408S, K417N, N440K, L452R, S477N, T478K, E484A, F486V, Q493R, Q498R, N501Y, Y505H, D614G, H655Y, N679K, P681H, N764K, D796Y, Q954H, N969K
**BQ.1**	T19I, Δ24-26, A27S, Δ69-70, G142D, Δ144, V213G, G339D, S371F, S373P, S375F, T376A, D405N, R408S, K417N, N440K, K444T, L452R, N460K, S477N, T478K, E484A, F486V, Q498R, N501Y, Y505H, D614G, H655Y, N679K, P681H, N764K, D796Y, Q954H, N969K
**BQ.1.1**	T19I, Δ24-26, A27S, Δ69-70, G142D, V213G, G339D, R346T, S371F, S373P, S375F, T376A, D405N, R408S, K417N, N440K, K444T, L452R,

	N460K, S477N, T478K, E484A, F486V, Q498R, N501Y, Y505H, D614G, H655Y, N679K, P681H, N764K, D796Y, Q954H, N969K
**XBB.1.5**	T19I, Δ24-26, A27S, V83A, G142D, Δ144, H146Q, Q183E, V213E, G252V, G339H, R346T, L368I, S371F, S373P, S375F, T376A, D405N, R408S, K417N, N440K, V445P, G446S, N460K, S477N, T478K, E484A, F486P, F490S, Q498R, N501Y, Y505H, D614G, H655Y, N679K, P681H, N764K, D796Y, Q954H, N969K

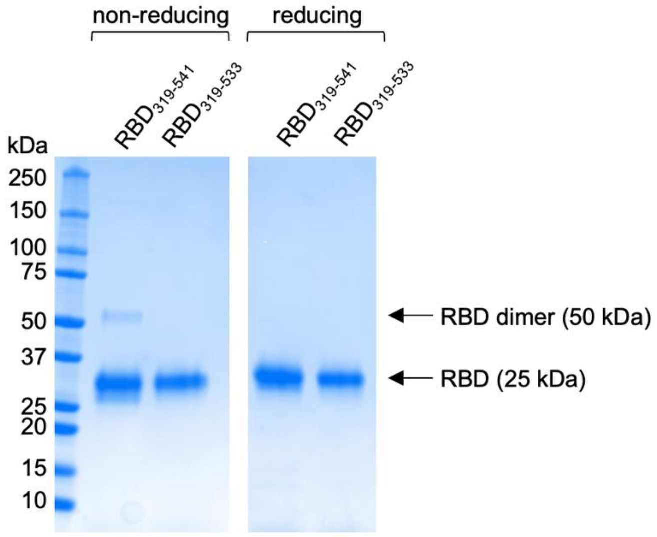

Figure S1. Removing the C-terminal cysteine from RBD substantially reduces dimer formation. Reducing and non-reducing SDS-PAGE gel of the RBD of Wuhan Hu-1 SARS-CoV-2 (GenBank MN908947.3) containing either residues 319-541 or residues 319-533 with one fewer cysteine residue.

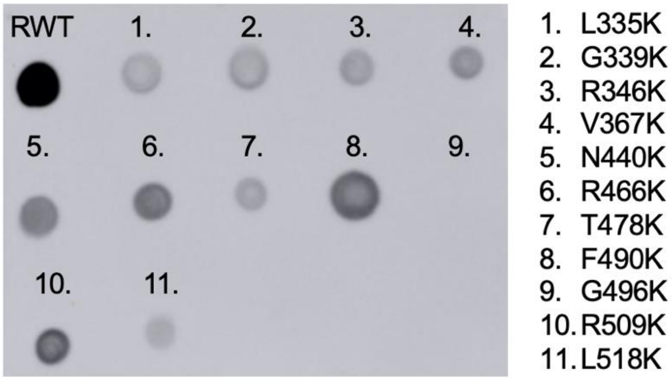

Figure S2. Expression levels of wild-type RBD and RBD variants containing lysine substitutions. A representative dot-blot is shown to compare the expression levels of wild-type RBD (RWT) and RBD variants from small-scale expressions. Supernatants from each cell culture of lysine-substituted RBDs (5 µL) were directly pipetted onto 0.2-µm nitrocellulose membranes before blocking and incubation with mAb S2X259 and detection with HRP-conjugated rabbit anti-human IgG. The blot was imaged on a GE Amersham Imager 600 and analyzed with Fiji (ImageJ v.2.1.0). The 7 lysine substitutions that were chosen to create R7K and their relative expression-level compared to RWT are: #3 R346K (59%), #4 V367K (47%), #5 N440K (163%), #6 R466K (104%), #7 T478K (38%), #8 F490K (142%), #11 L518K (33%).

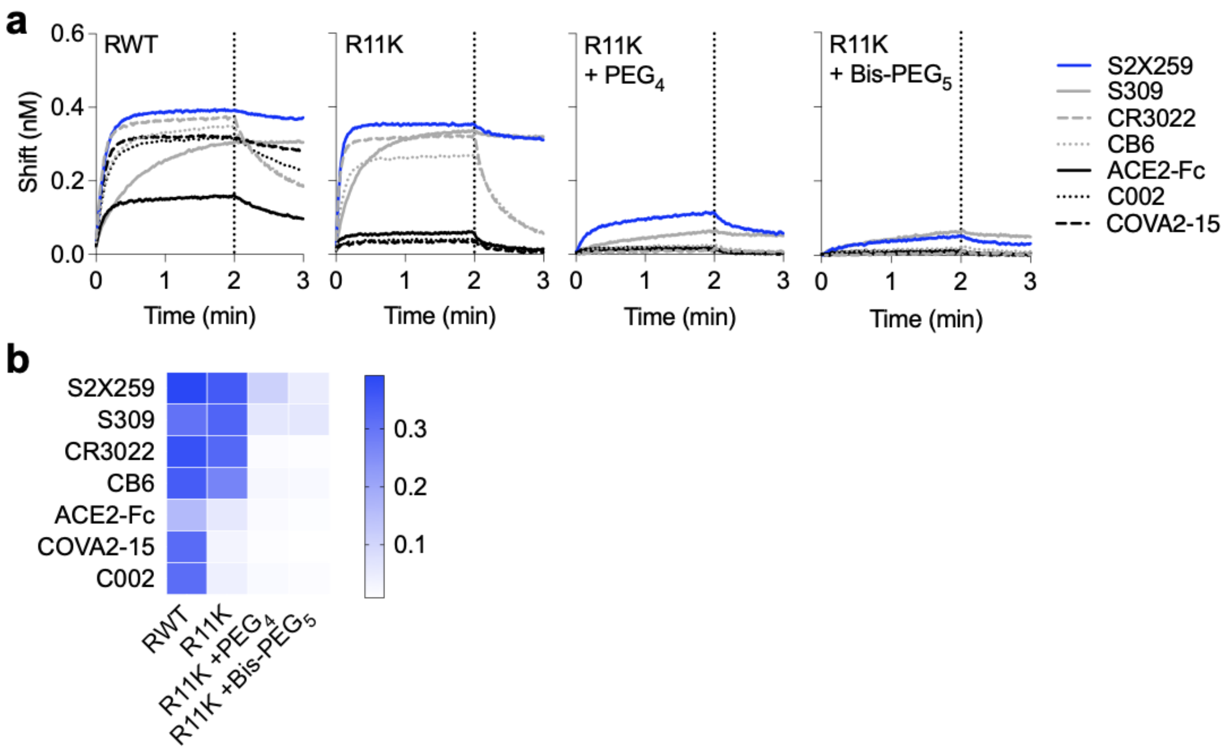

Figure S3. Modification of R11K prior to protection severely decreases binding to S2X259. R11K was reacted with NHS-PEG_4 or Bis-NHS-PEG_5 in solution, omitting the PMD protection step in which R11K is bound to an S2X259-conjugated resin. After reaction for 30 min, excess PEG moieties were removed by size-exclusion chromatography. **a**, BLI binding curves of RWT, R11K, and the purified PEGylated proteins (R11K+PEG_4 and R11K+Bis-PEG_5) binding to a panel of RBD-directed antibodies, including S2X259, S309 and CR3022. **b**, BLI association amplitude of each antibody binding to RWT, R11K, R11K+ PEG_4, R11K+Bis-PEG_5, as shown in **a**.

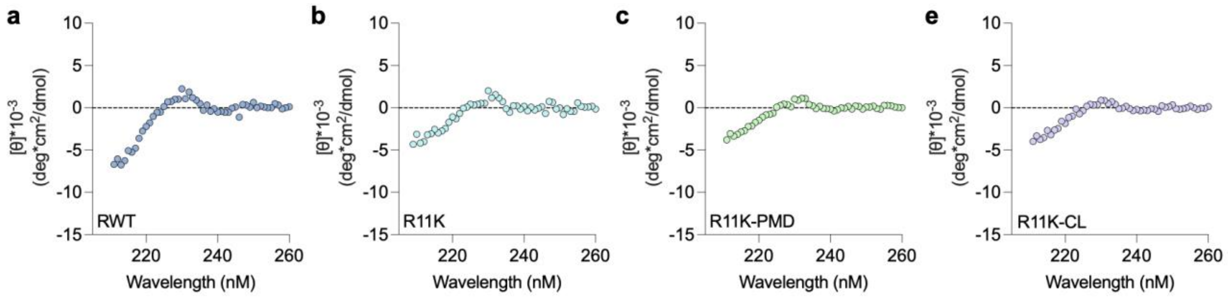

Figure S4. Circular dichroism (CD) spectroscopy of RWT, R11K, R11K-PMD and R11K-CL. CD spectra of filtered proteins in PBS at a concentration of 0.1-0.3 mg/mL are shown for RWT (**a**), R11K (**b**), R11K-PMD (**c**), R11K-CL (**d**).

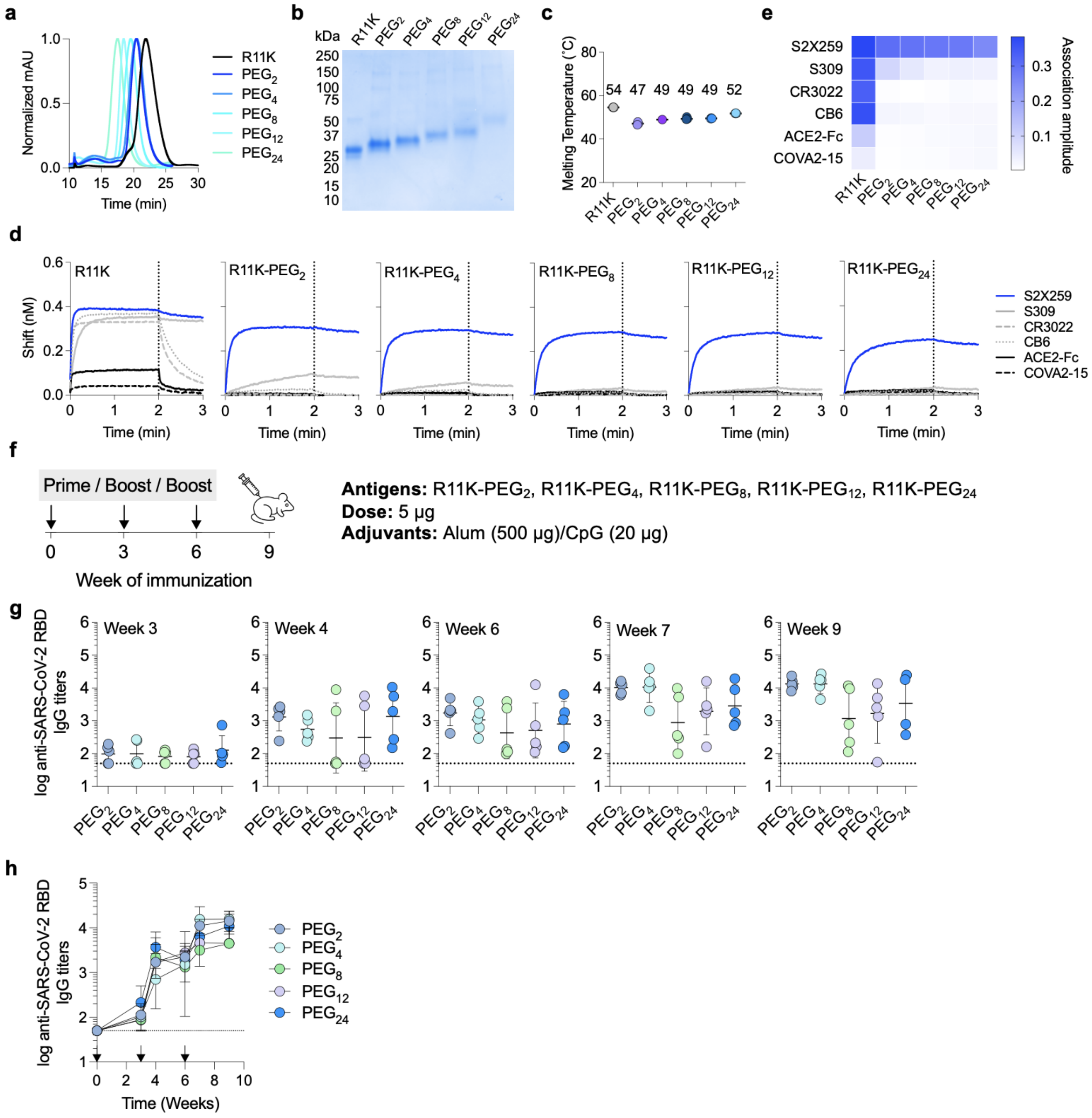

Figure S5. Antigenicity of R11K modified with different PEG lengths. a, Normalized size-exclusion chromatographic traces of R11K and R11K variants modified with PEG chains of differing lengths (n = 2,4,8,12, or 24). **b**, SDS-PAGE analysis of R11K and PEG-modified R11K variants post-expression and purification via size-exclusion chromatography. **c**, Thermal melting temperature for R11K, R11K-PEG$_2$, R11K-PEG$_4$, R11K-PEG$_8$, R11K-PEG$_{12}$, and R11K-PEG$_{24}$ measured by differential scanning fluorimetry. Data are presented as mean ± standard deviation (n

= 3 replicates). **d**, Binding curves of RBD-directed antibodies from all four classes to R11K, R11K-PEG$_2$, R11K-PEG$_4$, R11K-PEG$_8$, R11K-PEG$_{12}$, and R11K-PEG$_{24}$. **e**, BLI association amplitude of each antibody binding to the R11K variants. Association was monitored for 2 min (dotted lines), after which dissociation was monitored for 1 min. **f**, Schematic of the mouse immunization with a three-dose regimen occuring on days 0, 21, and 42. Mice were immunized with 5 *ug* of antigen adjuvanted with alum/CpG (500 *ug*/20 *ug*) *via* intramuscular injection (n = 5 per group). **g**, Serum IgG titers to SARS-CoV-2 RBD at 3, 4, and 6 weeks post-boost 1 (the latter two corresponding to 1 and 3 weeks post-boost 2). Each circle represents an individual mouse and the mean titers are indicated with a dash. **h**, Serum IgG titers to SARS-COV-2 RBD over time. Horizontal dotted lines indicate the limits of quantification. Data are presented as geometric mean ± standard deviation.

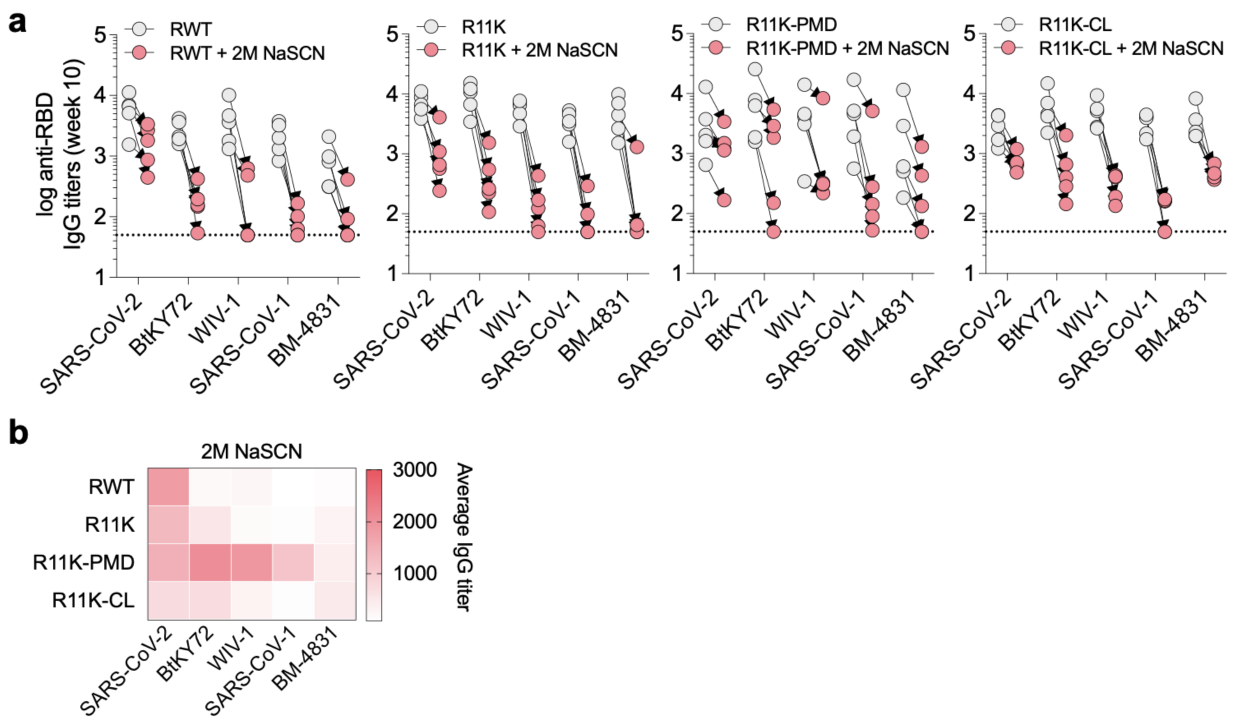

Figure S6. Antibody avidity assay. a, Serum IgG titers against different biotinylated RBDs in the presence or absence of treatment with 2M sodium thiocyanate (NaSCN). Each circle represents a single mouse. Horizontal dotted lines indicate the limit of quantitation. **b**, Average serum IgG titers of each immunization group to different RBDs post-treatment with 2M NaSCN presented as a heat map.

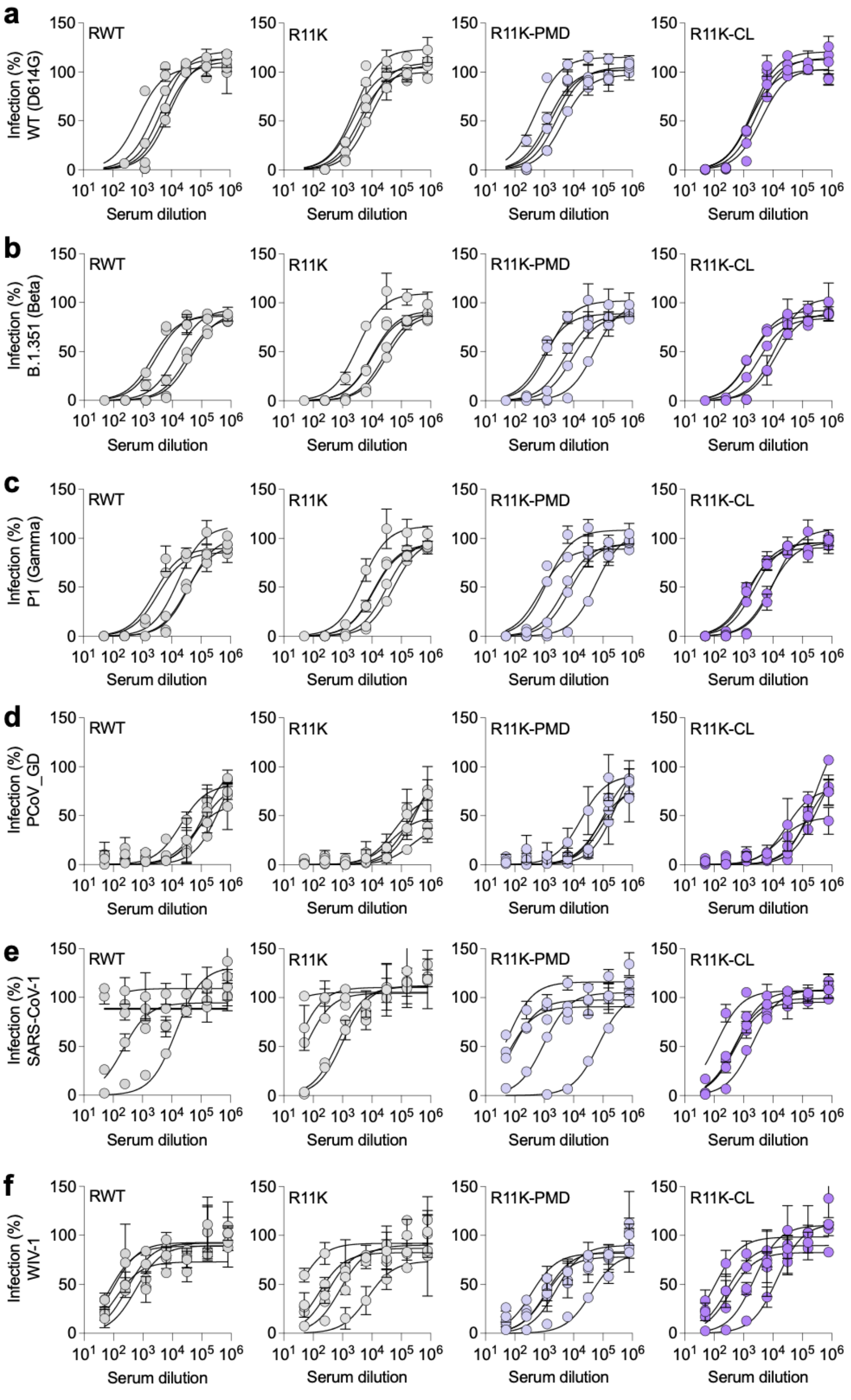

Figure S7. Neutralization of *Sarbecovirus* spike-pseudotyped lentiviruses. Antisera from mice immunized with either RWT, R11K, R11K-PMD, or R11K-CL were assessed on day 112 for neutralizing activity in HeLa cells overexpressing ACE2 and TMPRSS2. Neutralization

curves are shown for each immunization group against wild-type SARS-CoV-2 (D614G) (**a**), SARS-CoV-2 Beta (**b**), SARS-CoV-2 Gamma (**c**), pangolin coronavirus PCoV_GD (**d**), SARS-CoV-1 (**e**), and WIV-1 (**f**). Data are presented as mean ± standard deviation (*n*=2 technical duplicates). Each curve represents a single mouse.

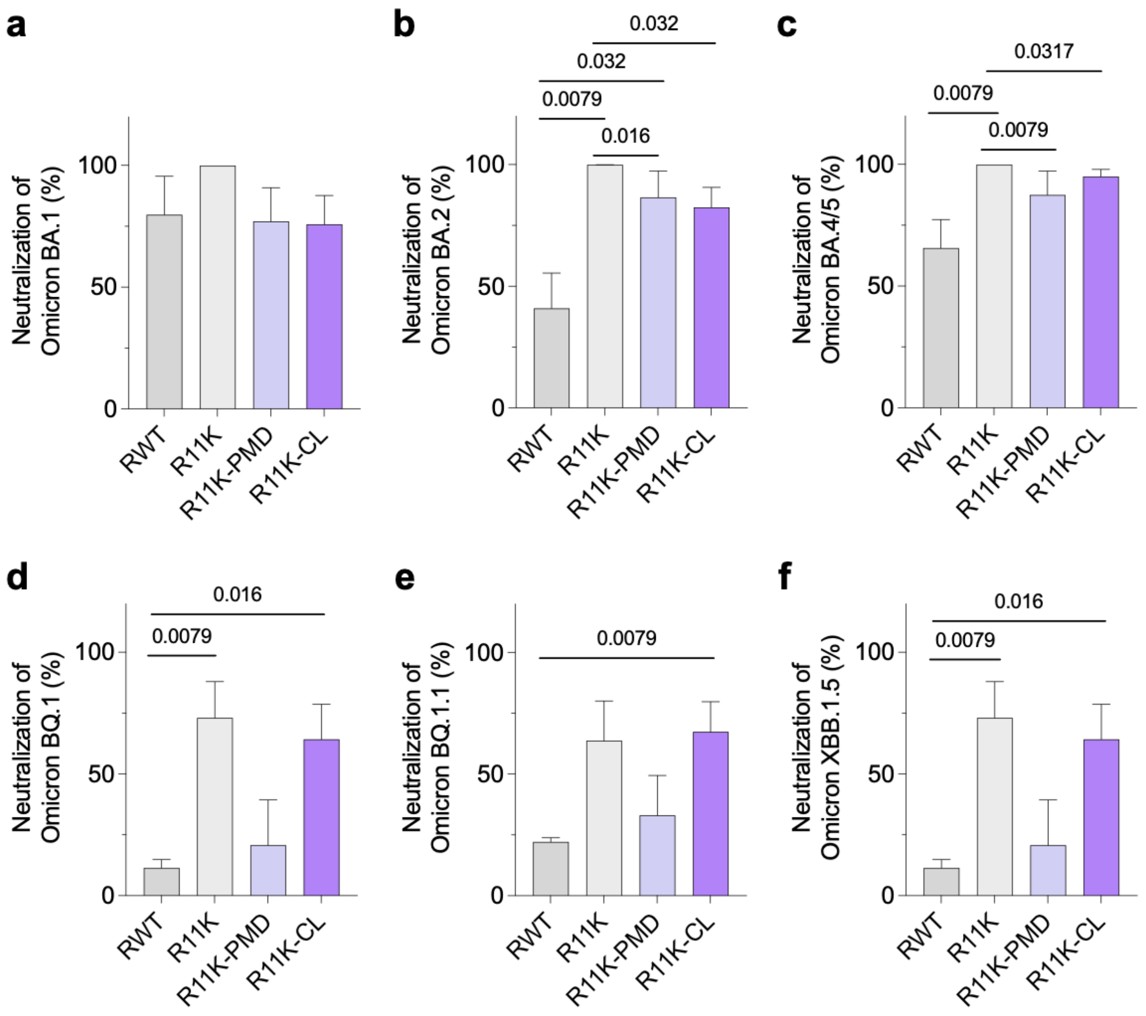

Figure S8. Neutralization of SARS-CoV-2 Omicron variants at a single serum dilution point. Antisera from mice immunized with either RWT, R11K, R11K-PMD, or R11K-CL was assessed on day 112 at a serum dilution of 1/160 for neutralizing activity against different SARS-CoV-2 Omicron pseudotyped lentiviruses in HeLa cells overexpressing ACE2 and TMPRSS2. Neutralizing activity of the five mice in each immunization group against Omicron BA.1 (**a**), Omicron BA.2 (**b**), Omicron BA.4/5 (**c**), Omicron BQ.1 (**d**), Omicron BQ.1.1 (**e**), and XBB.1.5 (**f**) was averaged and plotted. Comparisons of two groups were performed using the two-tailed Mann-Whitney U test. *P* values of 0.05 or less were considered significant and indicated.

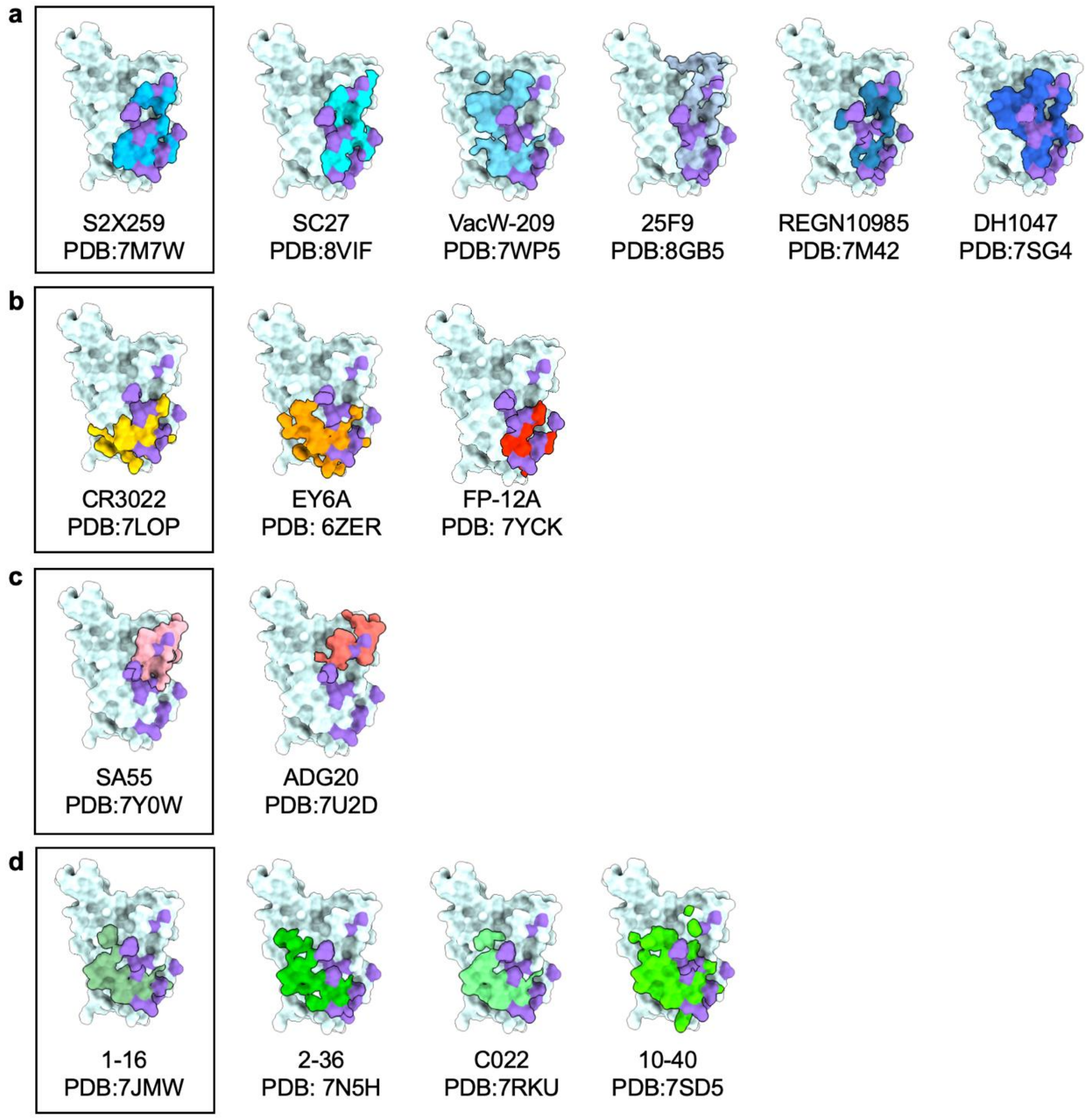

Figure S9. Structural footprints of class 4 antibodies on the SARS-CoV-2 RBD. The cryptic face of the SARS-CoV-2 RBD (PDB ID: 7M7W) is shown with the amino acid substitutions of R-KO4 colored in purple and the footprints of each antibody binding site outlined in black and highlighted by a different color. The structural footprints were made by highlighting residues on the RBD <5Å from the heavy or light chain of each antibody. Class 4 antibodies are grouped based on binding site similarity with S2X259 (**a**), CR3022 (**b**), SA55 (**c**), or 1-16 (**d**).

Representative antibodies from each category used in Figure 4b to validate R-KO4 are surrounded by a black box. Structures were prepared using UCSF-ChimeraX 1.5.

Supplementary Tables.

Table S1. Dissociation constants (k_{off}) for the binding of RBD-targeting antibodies to RBD immunogens RWT, R11K, R11K-PMD, and R11K-CL.

	k_{off} (10^{-3} s^{-1})			
Binding partner	**RWT**	**R11K**	**R11K-PMD**	**R11K-CL**
**S2X259**	1.1	2.1	0.85	1.0
**S309**	0.000000013	0.14	1.9	2.1
**CR3022**	16	59	64	63
**CB6**	13	37	n.d.	n.d.
**ACE2-Fc**	11	93	n.d.	n.d.
**COVA2-15**	2.5	83	n.d.	n.d.
**C002**	6.2	100.	n.d.	n.d.

n.d. = not determined
Dissociation constants calculated in GraphPad Prism 9.5.1 from the binding curves shown in Figure 2f.

Antibody Sequences

Antibody	PDB/EMD code	Sequence of variable regions
S2X259	7RA8	HC:QVQLVQSGAEVKKPGSSVKVSCKASGGIFNTYTISWVRQAP GQGLEWMGRIILMSGMANYAQKIQGRVTITADKSTSTAYMELTS LRSDDTAVYYCARGFNGNYYGWGDDDAFDIWGQGTLVTVYS<br><br>LC:QTVLTQPPSVSGAPGQRVTISCTGSNSNIGAGYDVHWYQQL PGTAPKLLICGNSNRPSGVPDRFSGSKSGTSASLAITGLQAEDE ADYYCQSYDSSLSGPNWVFGGGTKLTVL

2-15	22061	HC:QVQLLESGGGLVQPGGSLRLSCAASGFTFSSYAMSWVRQAP<br>GKGLEWASAISGSGGSTYYADSVEGRFTISRDNSKNTLYLQMNS<br>LRAEDTAVYYCAKDTGYCGDDCYIKLIRGGPDYWGQGTLVTVSS<br><br>LC:DIVMTQSPLSLPVTLGQPASISCRSSQSLVYSDGNTFLNWF<br>QQRPGQSPRRLIYQVSNRDSGVPDRFSGSGSGTDFTLKISRVEA<br>EDVGVYYCMQGTHWPRTFGQGTKLEIK
C002	7K8S	HC:EVQLVESGGGVVQPGRSLRLSCAASGFTFSIYGMHWVRQAP<br>GKGLEWVAVISYDGSNKYYADSVKGRFTISRDNSKNTLYLQMNS<br>LRAEDTAVYYCAKEGRPSDIVVVVAFDYWGQGTLVTVSS<br><br>LC:DIQLTQSPSSLSASVGDRVTITCRASQSISSYLNWYQQKPG<br>KAPKLLIYAASSLQSGVPSRFSGSGSGTDFTLTISSLQPEDFAT<br>YYCQQSYSTPRTFGQGTKVEIK
S309	6WPS	HC:QVQLVQSGAEVKKPGASVKVSCKASGYPFTSYGISWVRQAP<br>GQGLEWMGWISTYNGNTNYAQKFQGRVTMTTDTSTTTGYMELRR<br>LRSDDTAVYYCARDYTRGAWFGESLIGGFDNWGQGTLVTVSS<br><br>LC:EIVLTQSPGTLSLSPGERATLSCRASQTVSSTSLAWYQQKP<br>GQAPRLLIYGASSRATGIPDRFSGSGSGTDFTLTISRLEPEDFA<br>VYYCQQHDTSLTFGGGTKVEIK

CR3022	7L0P	HC:QMQLVQSGTEVKKPGESLKISCKGSGYGFITYWIGWVRQMP GKGLEWMGIIYPGDSETRYSPSFQGQVTISADKSINTAYLQWSS LKASDTAIYYCAGGSGISTPMDVWGQGTTVTV<br><br>LC:DIQLTQSPDSLAVSLGERATINCKSSQSVLYSSINKNYLAW YQQKPGQPPKLLIYWASTRESGVPDRFSGSGSGTDFTLTISSLQ AEDVAVYYCQQYYSTPYTFGQGTKVEIK
CB6	7C01	HC:EVQLVESGGGLVQPGGSLRLSCAASGFTVSSNYMSWVRQAP GKGLEWVSVIYSGGSTFYADSVKGRFTISRDNSMNTLFLQMNSL RAEDTAVYYCARVLPMYGDYLDYWGQGTLVTVSS<br><br>LC:DIVMTQSPSSLSASVGDRVTITCRASQSISRYLNWYQQKPG KAPKLLIYAASSLQSGVPSRFSGSGSGTDFTLTISSLQPEDFAT YYCQQSYSTPPEYTFGQGTKLEIK
SA55	7Y0W	HC:QVQLVQSGAEVKKPGSSVKVSCKASGGTFRSHVISWVRQAP GQGLEWMGGFIPLFGTTIYAQAFQGRVMISADESTSTAYMELSS LRSEDTAVYFCARLFPNGDPNSPEDGFDIWGQGTLVTVSS<br><br>LC:DIQMTQSPSSLSASVGDRVTITCQASQDIGNYLNWYQQKPG KAPKLLIYDASHLETGVPSRFSGSGSGTDFTFTISSLQPEDIAT YYCQRYDDLPSYTFGQGTKVEIK

C022	7RKU	HC:QVQLQESGPGLVKPSETLSVTCTVSGGSISSSRYYWGWIRQ<br>PPGKGLEWIGSIYYSGSTYYNPSLKSRVTISVDTSKNQFSLKLS<br>SVTAADTAVYYCARHAAAYYDRSGYYFIEYFQHWGQGTLVTVSS<br><br>LC:DIQMTQSPSTLSASVGDSVTITCRASQSISSWLAWYQQKPG<br>KAPKLLIYKASSLESGVPSRFSGSGSGTEFTLTISSLQPDDFAT<br>YYCQQYNNYRYTFGQGTKLEIK
2-36	7N5H	HC:QVQLQESGPGLVKPSETLSLTCTVSGGSVSSSNYYWSWIRQ<br>PPGKGLEWIGYMYYSGSTKYNPSLKSRVTISVDTSKNQFSLKLS<br>SVTAADTAVYYCAREVYYYDRSGYYASDGFDIWGQGTMVTVSS<br><br>LC:EIVLTQSPGTLSLSPGERATLSCRASQSVSSSYLAWYQQKP<br>GQAPRLLIYGASSRATGIPDRFSGSGSGTDFTLTISRLEPEDFA<br>VYYCQQYGSSPQTFGQGTKVEIK
1-16	7JMW	HC:QVQLVQSGAEVKKPGASVKVSCKASGYTFTSYYMHWVRQAP<br>GQGLEWMGIINSSGGSTSYAQKFQGRVTMTRDTSTSTVYMELSS<br>LRSEDTAVYYCARPPRNYYDRSGYYQRAEYFQHWGQGTLVTVSS<br><br>LC:DIQLTQSPSSLSASVGDRVTITCQASQDISNYLNWYQQRPG<br>KAPKLLIYDASNLETGVPSRFSGSGSGTDFTFTISSLQPEDIAT<br>YYCQQYDNPPLTFGGGTKLEIK

RBD Sequences

Hexa-histidine tag (underlined residues)

Avi-Tag (boldfaced residues)

Fc region (italicized residues)

TEV site (boldface italicized residues)

SARS-CoV-2 RBD (RWT)

RVQPTESIVRFPNITNLCPFGEVFNATRFASVYAWNRKRISNCVADYSVLYNSASFSTFKCYGV

SPTKLNDLCFTNVYADSFVIRGDEVRQIAPGQTGKIADYNYKLPDDFTGCVIAWNSNNLDSKVG

GNYNYLYRLFRKSNLKPFERDISTEIYQAGSTPCNGVEGFNCYFPLQSYGFQPTNGVGYQPYRV

VVLSFELLHAPATVCGPKKSTNLGHHHHHHG**GLNDIFEAQKIEWHE**

R7K

RVQPTESIVRFPNITNLCPFGEVFNATKFASVYAWNRKRISNCVADYSKLYNSASFSTFKCYGV

SPTKLNDLCFTNVYADSFVIRGDEVRQIAPGQTGKIADYNYKLPDDFTGCVIAWNSNKLDSKVG

GNYNYLYRLFRKSNLKPFEKDISTEIYQAGSKPCNGVEGFNCYKPLQSYGFQPTNGVGYQPYRV

VVLSFELKHAPATVCGPKKSTNLGHHHHHH

R11K

RVQPTESIVRFPNITNLCPFGEVFNATKFASVYAWNRKRISNCVADFSKLYNSASFSTFKCYGV

SPTKLNDLCWTNIYADSFVIRGDEVRQIAPGQTGKIADYNYKLPDDFTGCVIAWNSNKLDSKVG

GNYNYKYRLFRKSNLKPFEKDISTEIYQAGSKPCNGKEGFNCYKPLQSYGFKPTNGVGYQPYRV

VVLSFELKHAPKTVCGPKKSTNLGHHHHHH

R12K

RVQPTESIVRFPNITKLCPFGEVFNATKFASVYAWNRKRISNCVADFSKLYNSASFSTFKCYGV

SPTKLNDLCWTNIYADSFVIRGDEVRQIAPGQTGKIADYNYKLPDDFTGCVIAWNSNKLDSKVG

GNYNYKYRLFRKSNLKPFEKDISTEIYQAGSKPCNGKEGFNCYKPLQSYGFKPTNGVGYQPYRV

VVLSFELKHAPKTVCGPKKSTNLGHHHHHH

ACE2-Fc

STIEEQAKTFLDKFNHEAEDLFYQSSLASWNYNTNITEENVQNMNNAGDKWSAFLKEQSTLAQM

YPLQEIQNLTVKLQLQALQQNGSSVLSEDKSKRLNTILNTMSTIYSTGKVCNPDNPQECLLLEP

GLNEIMANSLDYNERLWAWESWRSEVGKQLRPLYEEYVVLKNEMARANHYEDYGDYWRGDYEVN

GVDGYDYSRGQLIEDVEHTFEEIKPLYEHLHAYVRAKLMNAYPSYISPIGCLPAHLLGDMWGRF

WTNLYSLTVPFGQKPNIDVTDAMVDQAWDAQRIFKEAEKFFVSVGLPNMTQGFWENSMLTDPGN

VQKAVCHPTAWDLGKGDFRILMCTKVTMDDFLTAHHEMGHIQYDMAYAAQPFLLRNGANEGFHE

AVGEIMSLSAATPKHLKSIGLLSPDFQEDNETEINFLLKQALTIVGTLPFTYMLEKWRWMVFKG

EIPKDQWMKKWWEMKREIVGVVEPVPHDETYCDPASLFHVSNDYSFIRYYTRTLYQFQFQEALC

QAAKHEGPLHKCDISNSTEAGQKLFNMLRLGKSEPWTLALENVVGAKNMNVRPLLNYFEPLFTW

LKDQNKNSFVGWSTDWSPYAD***ENLYFQG***GSGG*DKTHTCPPCPAPELLGGPSVFLFPPKPKDTLM*

ISRTPEVTCVVVDVSHEDPEVKFNWYVDGVEVHNAKTKPREEQYNSTYRVVSVLTVLHQDWLNG

KEYKCKVSNKALPAPIEKTISKAKGQPREPQVYTLPPSRDELTKNQVSLTCLVKGFYPSDIAVE

WESNGQPENNYKTTPPVLDSDGSFFLYSKLTVDKSRWQQGNVFSCSVMHEALHNHYTQKSLSLS

PGK

R-KO4

RVQPTESIVRFPNITNLCPFGEVFNATRFASVYAWNRKRISNCVADFSVLANSESFSHFNCYGV

EPYKLNDLCFTNVYADSFVIRGDEVAQIAPGQTGKIADYNYKLPDDFTGCVIAWNSNNLDSKVG

GNYNYLYRLFRKSNLKPFERDISTEIYQAGSTPCNGVEGFNCYFPLQSYGFQPTNDVDYQPYRV

VVLSFELLHAPATVCGPKKSTNLGHHHHHHG**GLNDIFEAQKIEWHE**
